# 2,2′-[Imidazolidine-1,3-diylbis(methyl­ene)]diphenol

**DOI:** 10.1107/S1600536811053748

**Published:** 2011-12-17

**Authors:** Augusto Rivera, Luz Stella Nerio, Jaime Ríos-Motta, Karla Fejfarová, Michal Dušek

**Affiliations:** a Departamento de Química, Universidad Nacional de Colombia, Ciudad Universitaria, Bogotá, Colombia; b Institute of Physics ASCR, v.v.i., Na Slovance 2, 182 21 Praha 8, Czech Republic

## Abstract

In the title mol­ecule, C_17_H_20_N_2_O_2_, the imidazolidine ring adopts a twist conformation. The mean plane through the five atoms of the imidazolidine ring makes dihedral angles of 70.18 (4) and 74.14 (4)° with the planes of the two aromatic rings. The dihedral angle between the benzene rings is 53.11 (5)°. Both phenol –OH groups form intra­molecular hydrogen bonds to the N atoms, with graph-set motif *S*(6). In the crystal, pairs of O—H⋯O hydrogen bonds link the mol­ecules into dimers with *R*_4_^4^(18) ring motifs. The crystal packing is further stabilized by C—H⋯O and weak C—H⋯π inter­actions.

## Related literature

For a related structure, see: Rivera *et al.* (2011 ▶). For the preparation of the title compound, see: Rivera *et al.* (1993 ▶). For ring conformations, see Cremer & Pople (1975 ▶). For hydrogen-bond graph-set nomenclature, see: Bernstein *et al.* (1995 ▶). For details of hydrogen bonding in Mannich bases, see: Koll *et al.* (2006 ▶); Filarowski *et al.* (1997 ▶).
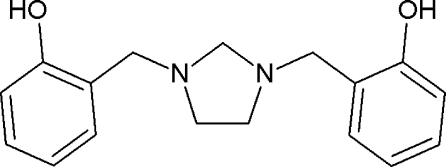


## Experimental

### 

#### Crystal data

C_17_H_20_N_2_O_2_
*M* *_r_* = 284.4
Monoclinic, 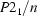

*a* = 9.6541 (6) Å
*b* = 9.5198 (11) Å
*c* = 16.0007 (19) Å
β = 97.321 (7)°
*V* = 1458.6 (3) Å^3^
*Z* = 4
Cu *K*α radiation
μ = 0.68 mm^−1^
*T* = 120 K
0.56 × 0.46 × 0.35 mm

#### Data collection

Agilent Xcalibur diffractometer with an Atlas (Gemini ultra Cu) detector
Absorption correction: multi-scan (*CrysAlis PRO*; Agilent, 2010 ▶) *T*_min_ = 0.684, *T*_max_ = 1
16045 measured reflections
2592 independent reflections
2323 reflections with *I* > 3σ(*I*)
*R*_int_ = 0.029

#### Refinement

*R*[*F* ^2^ > 2σ(*F* ^2^)] = 0.032
*wR*(*F* ^2^) = 0.102
*S* = 1.84
2592 reflections
197 parameters
2 restraints
H atoms treated by a mixture of independent and constrained refinement
Δρ_max_ = 0.18 e Å^−3^
Δρ_min_ = −0.16 e Å^−3^

### 

Data collection: *CrysAlis PRO* (Agilent, 2010 ▶); cell refinement: *CrysAlis PRO*; data reduction: *CrysAlis PRO*; program(s) used to solve structure: *SIR2002* (Burla *et al.*, 2003 ▶); program(s) used to refine structure: *JANA2006* (Petříček *et al.*, 2006 ▶); molecular graphics: *DIAMOND* (Brandenburg & Putz, 2005 ▶); software used to prepare material for publication: *JANA2006*.

## Supplementary Material

Crystal structure: contains datablock(s) global, I. DOI: 10.1107/S1600536811053748/bt5752sup1.cif

Structure factors: contains datablock(s) I. DOI: 10.1107/S1600536811053748/bt5752Isup2.hkl

Supplementary material file. DOI: 10.1107/S1600536811053748/bt5752Isup3.cml

Additional supplementary materials:  crystallographic information; 3D view; checkCIF report

## Figures and Tables

**Table 1:** Hydrogen-bond geometry (Å, °) *Cg*3 is the centroid of the C12–C17 benzene rings.

*D*—H⋯*A*	*D*—H	H⋯*A*	*D*⋯*A*	*D*—H⋯*A*
O1—H1*o*⋯N1	0.88 (1)	1.83 (1)	2.6394 (13)	152 (2)
O2—H2*o*⋯N2	0.88 (1)	1.84 (1)	2.6557 (13)	154 (2)
O1—H1*o*⋯O1^i^	0.88 (1)	2.60 (2)	3.0232 (12)	111 (1)
C11—H11*a*⋯O1^i^	0.96	2.58	3.4961 (15)	159
C14—H14⋯O1^ii^	0.96	2.50	3.4561 (15)	172
C6—H6⋯*Cg*3^iii^	0.96	2.97	3.7868 (14)	143
C10—H10*b*⋯*Cg*3^ii^	0.96	2.83	3.6718 (13)	148

Symmetry codes: (i) 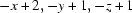; (ii) 
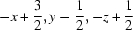
; (iii) 
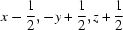
.

**Fig. 1.**
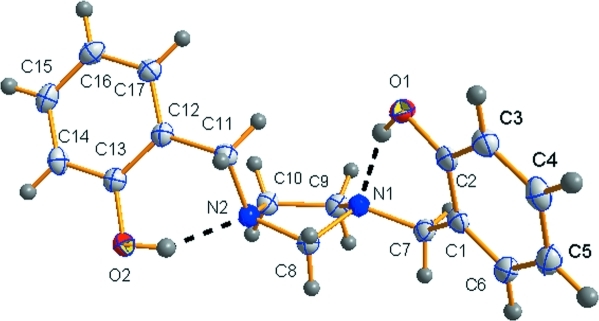
A view of (I) with the numbering scheme. Displacement ellipsoids are drawn at the 50% probability level.

**Fig. 2.**
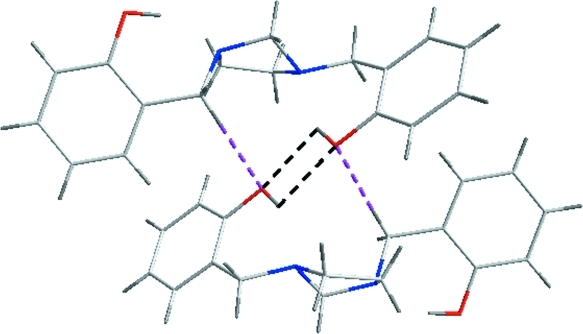
Dimer formation of the title compound by a R44(18) ring motif.

**Fig. 3.**
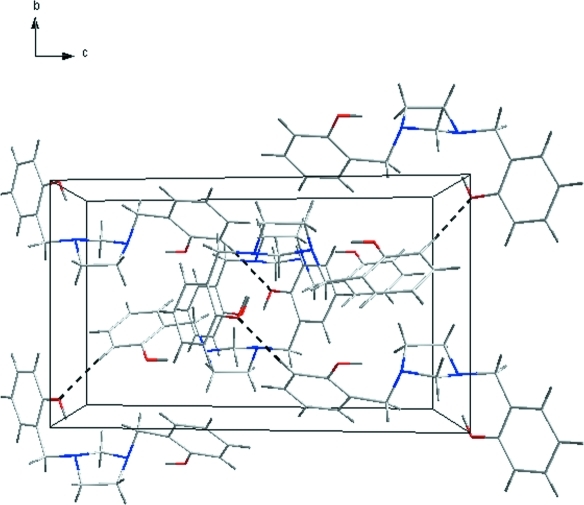
Packing of the molecules of the title compound view along the b axis.

## supplementary crystallographic information

### Comment

The study of intramolecular hydrogen bonds is interesting because of the high thermodynamic and structural stability of these systems. Intramolecularly hydrogen-bonded systems with direct coupling between acid and base centers reveal properties which make them valuable materials for practical use (Koll *et al.*, 2006). Of the various different bifunctional intramolecularly hydrogen bonded compounds, *ortho*-Mannich bases are of special interest due to the presence of an electronic coupling between the proton donating and proton accepting groups through the aromatic ring which potentially affords structural consequences (Filarowski *et al.*, 1997). We report here the crystal structure analysis of the title compound, (I).

The asymmetric unit of title compound (I) contains one molecule which have no internal symmetry (Fig 1). In (I), Fig. 1, the imidazolidine ring is twisted about the N1—C8 bond as seen in the puckering parameters Q(2) = 0.4126 (12) Å and φ = 20.02 (17) ° (Cremer & Pople, 1975). The mean plane of imidazolidine ring defined by N2, C9 and C10 makes a dihedral angle of 67.326 (56)° and 76.528 (47)° with the two pendant aromatic rings, C1—C6 and C12—C17 respectively. The dihedral angle between the phenyl rings is 53.134 (39)°. Both N atoms serves as hydrogen-bond acceptors to the phenolic OH group forming two intramolecular O—H···N hydrogen bond with graph-set motif S(6).

The molecular packing in (I) facilitates reciprocal O1—H1···O1 and C11—H11*a*···O1 interactions which join two crystallographically independent molecules into a dimer (Figure 2) forming an *R*_4_^4^(18) ring motif. The dimers are further connected *via* C14—H14···O1 hydrogen bonds and weak C—H···π, forming chains along [111] (Figure 3).

### Experimental

For the originally reported synthesis, see: Rivera *et al.* (1993)

### Refinement

All H atoms bonded to carbon atoms were positioned geometrically and treated as riding on their parent atoms. The hydroxyl H atoms were found in difference Fourier maps ant their coordinates were refined with a distance restraint of 0.87 Å with σ of 0.01. All H atoms were refined with  displacement coefficients *U*_iso_(H) set to 1.5*U*_eq_(O) for hydroxyl groups and to 1.2Ueq(C) for the –CH– and CH_2_– groups.

### Crystal data

**Table:**

C_17_H_20_N_2_O_2_	*F*(000) = 608
*M**_r_* = 284.4	*D*_x_ = 1.295 Mg m^−^^3^
Monoclinic, *P*2_1_/*n*	Cu *K*α radiation, λ = 1.5418 Å
Hall symbol: -P 2yn	Cell parameters from 9734 reflections
*a* = 9.6541 (6) Å	θ = 2.8–66.9°
*b* = 9.5198 (11) Å	µ = 0.68 mm^−^^1^
*c* = 16.0007 (19) Å	*T* = 120 K
β = 97.321 (7)°	Block, colourless
*V* = 1458.6 (3) Å^3^	0.56 × 0.46 × 0.35 mm
*Z* = 4	

### Data collection

**Table:**

Agilent Xcalibur diffractometer with an Atlas (Gemini ultra Cu) detector	2592 independent reflections
Radiation source: Enhance Ultra (Cu) X-ray Source	2323 reflections with *I* > 3σ(*I*)
mirror	*R*_int_ = 0.029
Detector resolution: 10.3784 pixels mm^-1^	θ_max_ = 67.1°, θ_min_ = 5.1°
Rotation method data acquisition using ω scans	*h* = −10→11
Absorption correction: multi-scan (*CrysAlis PRO*; Agilent, 2010)	*k* = −11→11
*T*_min_ = 0.684, *T*_max_ = 1	*l* = −18→18
16045 measured reflections	

### Refinement

**Table:**

Refinement on *F*^2^	H atoms treated by a mixture of independent and constrained refinement
*R*[*F*^2^ > 2σ(*F*^2^)] = 0.032	Weighting scheme based on measured s.u.'s *w* = 1/(σ^2^(*I*) + 0.0016*I*^2^)
*wR*(*F*^2^) = 0.102	(Δ/σ)_max_ = 0.008
*S* = 1.84	Δρ_max_ = 0.18 e Å^−^^3^
2592 reflections	Δρ_min_ = −0.16 e Å^−^^3^
197 parameters	Extinction correction: B-C type 1 Lorentzian isotropic (Becker & Coppens, 1974)
2 restraints	Extinction coefficient: 2300 (400)
74 constraints	

### Special details

**Table:**

Experimental. CrysAlisPro (Agilent, 2010) Empirical absorption correction using spherical harmonics, implemented in SCALE3 ABSPACK scaling algorithm.
Refinement. The refinement was carried out against all reflections. The conventional *R*-factor is always based on *F*. The goodness of fit as well as the weighted *R*-factor are based on *F* and *F*^2^ for refinement carried out on *F* and *F*^2^, respectively. The threshold expression is used only for calculating *R*-factors *etc*. and it is not relevant to the choice of reflections for refinement.The program used for refinement, Jana2006, uses the weighting scheme based on the experimental expectations, see _refine_ls_weighting_details, that does not force *S* to be one. Therefore the values of *S* are usually larger than the ones from the *SHELX* program.

### Fractional atomic coordinates and isotropic or equivalent isotropic displacement parameters (Å^2^)

**Table:**

	*x*	*y*	*z*	*U*_iso_*/*U*_eq_	
O1	0.88118 (9)	0.55808 (8)	0.54258 (5)	0.0258 (3)	
O2	0.64185 (9)	0.24108 (8)	0.20085 (5)	0.0266 (3)	
N1	0.82182 (10)	0.29372 (9)	0.50324 (6)	0.0216 (3)	
N2	0.75183 (10)	0.27736 (10)	0.36014 (6)	0.0230 (3)	
C1	0.73543 (12)	0.41864 (12)	0.62221 (7)	0.0220 (3)	
C2	0.78452 (12)	0.54925 (11)	0.59766 (7)	0.0223 (3)	
C3	0.73697 (13)	0.67284 (12)	0.63044 (7)	0.0263 (4)	
C4	0.64267 (13)	0.66779 (12)	0.68865 (7)	0.0286 (4)	
C5	0.59312 (13)	0.53957 (13)	0.71379 (7)	0.0293 (4)	
C6	0.63913 (13)	0.41633 (12)	0.67971 (7)	0.0260 (4)	
C7	0.79468 (12)	0.28403 (11)	0.59129 (7)	0.0234 (3)	
C8	0.69700 (12)	0.29649 (12)	0.44015 (7)	0.0234 (3)	
C9	0.89999 (13)	0.17320 (11)	0.47679 (7)	0.0258 (4)	
C10	0.86888 (13)	0.17560 (12)	0.38032 (7)	0.0261 (4)	
C11	0.80113 (12)	0.41147 (11)	0.32684 (7)	0.0234 (3)	
C12	0.84082 (12)	0.39145 (11)	0.23931 (7)	0.0220 (3)	
C13	0.75755 (12)	0.30847 (11)	0.18009 (7)	0.0226 (3)	
C14	0.79084 (13)	0.29425 (12)	0.09837 (7)	0.0260 (4)	
C15	0.90434 (13)	0.36558 (12)	0.07451 (7)	0.0286 (4)	
C16	0.98804 (13)	0.44846 (12)	0.13217 (8)	0.0282 (4)	
C17	0.95637 (12)	0.45896 (11)	0.21422 (7)	0.0241 (3)	
H3	0.769609	0.761889	0.612664	0.0316*	
H4	0.611303	0.753394	0.711789	0.0343*	
H5	0.527828	0.536104	0.754248	0.0352*	
H6	0.603597	0.327787	0.696268	0.0311*	
H7a	0.879717	0.260909	0.626476	0.0281*	
H7b	0.731088	0.208142	0.597016	0.0281*	
H8a	0.637442	0.218852	0.449563	0.0281*	
H8b	0.652961	0.386822	0.44087	0.0281*	
H9a	0.863665	0.08824	0.497922	0.031*	
H9b	0.99806	0.187851	0.493244	0.031*	
H10a	0.949631	0.208598	0.356872	0.0313*	
H10b	0.83935	0.083954	0.360322	0.0313*	
H11a	0.880577	0.444991	0.363629	0.0281*	
H11b	0.728563	0.480712	0.325292	0.0281*	
H14	0.735192	0.235123	0.058695	0.0312*	
H15	0.925546	0.357838	0.017685	0.0343*	
H16	1.066759	0.49779	0.115436	0.0338*	
H17	1.015526	0.514012	0.254456	0.0289*	
H1o	0.8869 (16)	0.4725 (11)	0.5220 (9)	0.0387*	
H2o	0.6533 (17)	0.2415 (17)	0.2562 (6)	0.0399*	

### Atomic displacement parameters (Å^2^)

**Table:**

	*U*^11^	*U*^22^	*U*^33^	*U*^12^	*U*^13^	*U*^23^
O1	0.0292 (5)	0.0224 (4)	0.0271 (4)	−0.0034 (3)	0.0088 (3)	−0.0011 (3)
O2	0.0273 (5)	0.0291 (4)	0.0231 (4)	−0.0045 (3)	0.0026 (3)	−0.0035 (3)
N1	0.0230 (5)	0.0218 (4)	0.0200 (5)	−0.0001 (4)	0.0032 (4)	−0.0009 (3)
N2	0.0263 (5)	0.0222 (5)	0.0209 (5)	−0.0016 (4)	0.0047 (4)	−0.0017 (3)
C1	0.0224 (6)	0.0249 (5)	0.0175 (5)	−0.0003 (4)	−0.0020 (4)	−0.0006 (4)
C2	0.0217 (6)	0.0264 (6)	0.0179 (5)	−0.0008 (4)	−0.0010 (4)	0.0003 (4)
C3	0.0309 (7)	0.0233 (6)	0.0238 (6)	0.0003 (4)	−0.0003 (5)	−0.0004 (4)
C4	0.0315 (7)	0.0281 (6)	0.0257 (6)	0.0049 (5)	0.0012 (5)	−0.0054 (4)
C5	0.0294 (7)	0.0362 (6)	0.0230 (6)	0.0020 (5)	0.0059 (5)	−0.0023 (5)
C6	0.0280 (7)	0.0280 (6)	0.0217 (6)	−0.0028 (5)	0.0030 (5)	0.0010 (4)
C7	0.0279 (6)	0.0222 (5)	0.0203 (6)	−0.0010 (4)	0.0037 (4)	0.0020 (4)
C8	0.0237 (6)	0.0249 (5)	0.0218 (6)	−0.0022 (4)	0.0036 (4)	−0.0013 (4)
C9	0.0292 (7)	0.0223 (5)	0.0266 (6)	0.0023 (4)	0.0060 (5)	−0.0001 (4)
C10	0.0309 (7)	0.0220 (5)	0.0258 (6)	0.0012 (4)	0.0057 (5)	−0.0023 (4)
C11	0.0273 (6)	0.0203 (5)	0.0223 (6)	−0.0023 (4)	0.0019 (5)	−0.0022 (4)
C12	0.0261 (6)	0.0183 (5)	0.0214 (6)	0.0030 (4)	0.0024 (4)	0.0007 (4)
C13	0.0235 (6)	0.0198 (5)	0.0245 (6)	0.0036 (4)	0.0027 (4)	0.0010 (4)
C14	0.0309 (7)	0.0248 (5)	0.0217 (6)	0.0053 (5)	0.0013 (5)	−0.0023 (4)
C15	0.0349 (7)	0.0294 (6)	0.0224 (6)	0.0083 (5)	0.0078 (5)	0.0037 (5)
C16	0.0292 (7)	0.0250 (6)	0.0313 (6)	0.0036 (5)	0.0079 (5)	0.0057 (4)
C17	0.0264 (6)	0.0193 (5)	0.0264 (6)	0.0017 (4)	0.0025 (5)	0.0018 (4)

### Geometric parameters (Å, °)

**Table:**

O1—C2	1.3654 (15)	C7—H7b	0.96
O1—H1o	0.883 (11)	C8—H8a	0.96
O2—C13	1.3653 (14)	C8—H8b	0.96
O2—H2o	0.879 (9)	C9—C10	1.5348 (16)
N1—C7	1.4684 (15)	C9—H9a	0.96
N1—C8	1.4703 (14)	C9—H9b	0.96
N1—C9	1.4650 (15)	C10—H10a	0.96
N2—C8	1.4578 (15)	C10—H10b	0.96
N2—C10	1.4922 (15)	C11—C12	1.5104 (16)
N2—C11	1.4849 (15)	C11—H11a	0.96
C1—C2	1.4044 (16)	C11—H11b	0.96
C1—C6	1.3890 (17)	C12—C13	1.4050 (15)
C1—C7	1.5120 (16)	C12—C17	1.3902 (17)
C2—C3	1.3898 (16)	C13—C14	1.3922 (17)
C3—C4	1.3837 (18)	C14—C15	1.3836 (18)
C3—H3	0.96	C14—H14	0.96
C4—C5	1.3890 (18)	C15—C16	1.3916 (16)
C4—H4	0.96	C15—H15	0.96
C5—C6	1.3902 (17)	C16—C17	1.3890 (18)
C5—H5	0.96	C16—H16	0.96
C6—H6	0.96	C17—H17	0.96
C7—H7a	0.96		
			
C2—O1—H1o	105.3 (10)	H8a—C8—H8b	114.38
C13—O2—H2o	104.0 (10)	N1—C9—C10	103.70 (9)
C7—N1—C8	115.40 (9)	N1—C9—H9a	109.4718
C7—N1—C9	112.92 (8)	N1—C9—H9b	109.4709
C8—N1—C9	102.89 (8)	C10—C9—H9a	109.4712
C8—N2—C10	103.93 (8)	C10—C9—H9b	109.4714
C8—N2—C11	112.12 (9)	H9a—C9—H9b	114.686
C10—N2—C11	111.54 (9)	N2—C10—C9	105.84 (9)
C2—C1—C6	118.51 (10)	N2—C10—H10a	109.4709
C2—C1—C7	120.24 (10)	N2—C10—H10b	109.4713
C6—C1—C7	121.09 (10)	C9—C10—H10a	109.4712
O1—C2—C1	121.18 (10)	C9—C10—H10b	109.4714
O1—C2—C3	118.51 (10)	H10a—C10—H10b	112.8697
C1—C2—C3	120.30 (11)	N2—C11—C12	110.80 (9)
C2—C3—C4	120.12 (11)	N2—C11—H11a	109.4718
C2—C3—H3	119.9399	N2—C11—H11b	109.4711
C4—C3—H3	119.9395	C12—C11—H11a	109.4708
C3—C4—C5	120.39 (11)	C12—C11—H11b	109.4713
C3—C4—H4	119.8038	H11a—C11—H11b	108.1129
C5—C4—H4	119.8053	C11—C12—C13	120.35 (10)
C4—C5—C6	119.27 (12)	C11—C12—C17	121.14 (9)
C4—C5—H5	120.3644	C13—C12—C17	118.45 (10)
C6—C5—H5	120.3649	O2—C13—C12	121.04 (10)
C1—C6—C5	121.38 (11)	O2—C13—C14	118.47 (10)
C1—C6—H6	119.3094	C12—C13—C14	120.49 (11)
C5—C6—H6	119.3085	C13—C14—C15	119.81 (10)
N1—C7—C1	112.45 (9)	C13—C14—H14	120.0949
N1—C7—H7a	109.4718	C15—C14—H14	120.0956
N1—C7—H7b	109.4717	C14—C15—C16	120.58 (11)
C1—C7—H7a	109.4708	C14—C15—H15	119.7101
C1—C7—H7b	109.4707	C16—C15—H15	119.7107
H7a—C7—H7b	106.316	C15—C16—C17	119.23 (11)
N1—C8—N2	104.07 (9)	C15—C16—H16	120.3866
N1—C8—H8a	109.471	C17—C16—H16	120.3878
N1—C8—H8b	109.4708	C12—C17—C16	121.40 (10)
N2—C8—H8a	109.4722	C12—C17—H17	119.2994
N2—C8—H8b	109.4714	C16—C17—H17	119.2985
			
?—?—?—?	?		

### Hydrogen-bond geometry (Å, °)

**Table:**

*Cg*3 is the centroid of the C12–C17 benzene rings.

**Table:**

*D*—H···*A*	*D*—H	H···*A*	*D*···*A*	*D*—H···*A*
O1—H1o···N1	0.88 (1)	1.83 (1)	2.6394 (13)	152.(2)
O2—H2o···N2	0.88 (1)	1.84 (1)	2.6557 (13)	154.(2)
O1—H1o···O1^i^	0.88 (1)	2.60 (2)	3.0232 (12)	111.(1)
C11—H11a···O1^i^	0.96	2.58	3.4961 (15)	159.
C14—H14···O1^ii^	0.96	2.50	3.4561 (15)	172.
C6—H6···Cg3^iii^	0.96	2.97	3.7868 (14)	143
C10—H10b···Cg3^ii^	0.96	2.83	3.6718 (13)	148

Symmetry codes: (i) −*x*+2, −*y*+1, −*z*+1; (ii) −*x*+3/2, *y*−1/2, −*z*+1/2; (iii) *x*−1/2, −*y*+1/2, *z*+1/2.
